# Stability Prediction of Multi-Factor Coupled Cast Iron Milling System Based on an Improved Full-Discretization Method

**DOI:** 10.3390/ma19122658

**Published:** 2026-06-20

**Authors:** Han Zhang, Minghui Li, Yan Xia

**Affiliations:** 1 School of Mechanical and Electronic Engineering, Shandong Jianzhu University, Jinan 250101, China; 2 China National Heavy Duty Truck Group Co., Ltd., Jinan 250000, China; lmh301739@163.com; 3 Shandong Key Laboratory of CNC Machine Tool Functional Components, School of Mechanical Engineering, Qilu University of Technology (Shandong Academy of Sciences), Jinan 250353, China; yxia@qlu.edu.cn; 4 Shandong Institute of Mechanical Design and Research, Jinan 250031, China

**Keywords:** cast iron machining, regenerative chatter, milling stability, full-discretization method, interpolation order optimization

## Abstract

Cast iron components are indispensable in aerospace and automotive systems, yet their milling operations are severely affected by regenerative chatter, which degrades machining quality and damages equipment. Although various chatter prediction methods have been reported, the optimal interpolation strategy of full-discretization methods (FDMs) for multi-factor coupled dynamic systems remains unclear. This study proposes an enhanced FDM to fill this research gap. A dynamic milling model accounting for regenerative effects, modal coupling and process damping is established, and an improved FDM based on Lagrange interpolation is further developed. A systematic single-factor analysis is carried out to examine the performance of 1st–4th-order interpolation for state, delay and periodic terms. Counter-intuitively, convergence analysis and stability lobe diagram (SLD) verification reveal that higher-order interpolation does not guarantee better performance. The optimal orders are identified as 2nd/3rd for state terms, 3rd for delay terms and 1st for periodic terms. Accordingly, the proposed 321-FDM (3rd-order state, 2nd-order delay, 1st-order periodic) exhibits higher accuracy and computational efficiency compared with benchmark methods, namely the semi-discretization method and Hermite-based 3rd-order FDM. Milling experiments on cast iron workpieces validate the established model and the 321-FDM, and the experimental stability thresholds agree well with numerical predictions. This work presents a validated, high-performance stability prediction tool for chatter avoidance in cast iron machining.

## 1. Introduction

**Figure 1:**
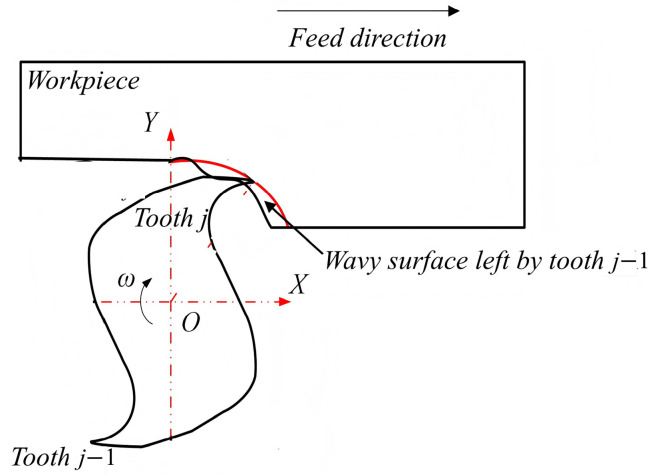
Schematic diagram of the regenerative chatter mechanism in milling.

Cast iron components, as core structural parts of industrial equipment, are widely employed in modern manufacturing. Nevertheless, cast iron workpieces are susceptible to chatter vibration during machining, as depicted in Figure 1, which degrades surface quality, reduces machining accuracy, induces abnormal tool wear, and even causes machine tool damage [1,2]. Milling stability prediction has become an effective means to avoid regenerative chatter. Reliable stability prediction requires the acquisition of dynamic parameters and cutting force coefficients of the machining system, as well as the implementation of dedicated stability prediction methods to solve the dynamic model and generate the corresponding SLD.

Global scholars have extensively explored milling stability prediction methods, which can be categorized into time-domain and frequency-domain approaches. Frequency-domain methods mainly include the zero-order analytical method (ZOA) [3] and the multi-frequency analytical method [4,5]. Time-domain approaches primarily cover the semi-discretization method (SDM) [6,7,8,9], full-discretization methods (FDMs) [10,11,12,13,14,15,16,17,18,19,20], time-domain finite element analysis (TFEA) [21,22], and the numerical integration method (NIM) [23,24,25,26,27,28,29,30,31]. Among these methods, FDMs and NIMs have become the dominant research hotspots in current studies.

Altintas and Budak proposed the ZOA in 1995 by approximating milling force coefficients using Fourier series [3]. Although feasible for general machining conditions, the ZOA exhibits inherent limitations in low-radial-immersion milling with small-diameter cutters. To tackle this issue, Budak and Altintas subsequently developed the multi-frequency prediction method (MFM) [4,5].

The SDM was originally established by Insperger and Stépán [6]. Insperger further refined this algorithm by weighting adjacent discrete delay terms to approximate the time-delay characteristics of milling systems [7,8]. This method essentially discretizes the tooth-passing period with uniform nodes, and its first-order and high-order numerical formulations were subsequently developed [9].

Based on the SDM framework, Ding proposed the full-discretization method (FDM), which achieves simultaneous discretization of system state variables and time-delay terms. First-order and second-order FDMs were established using first- and second-order Lagrange polynomials for state-terms, alongside linear interpolation for delay and periodic coefficients [10,11]. Comparative results revealed that the second-order FDM exhibits superior convergence performance compared with the first-order FDM and SDM. Tang et al. adopted second-order Lagrange polynomials to interpolate both state and delay terms, effectively improving the prediction accuracy of milling stability [12]. Qin et al. developed a unified interpolation strategy, where state, delay, and periodic terms are jointly approximated via second-order Lagrange polynomials, achieving simultaneous improvements in computational accuracy and efficiency [13]. Liu et al. constructed a third-order FDM utilizing Hermite polynomials for state term approximation, which outperformed conventional SDM and low-order FDMs in terms of convergence and computational efficiency [14]. Guo et al. utilized third-order Newton polynomials to interpolate state terms, and comparative analyses verified the superior performance of the third-order interpolation scheme [15]. Yang et al. adopted a hybrid interpolation strategy, where third-order Newton polynomials interpolate state terms and second-order Hermite polynomials interpolate delay terms, improving calculation accuracy and computational efficiency [16]. Ma et al. adopted cubic spline interpolation for state terms and third-order Newton polynomial interpolation for delay terms and proposed an optimized algorithm to improve computational efficiency [17]. Li et al. established a qualitative state-space model. The state and delay terms were discretized via linear interpolation, and time-varying terms were evaluated by numerical integration. Numerical examples verified its advantages in computational efficiency and numerical accuracy [18]. For high-order algorithm development, Zhou et al. proposed a fourth-order FDM based on fourth-order Lagrange polynomials [19], while Ozoegwu et al. developed fourth-order and fifth-order FDMs via least-squares approximation. Their study indicated that the fifth-order FDM cannot achieve higher accuracy than the fourth-order counterpart [20].

Bayly et al. proposed the TFEA, which distinguishes tool entry and exit behaviors into free- and forced-vibration regimes and enables high-precision stability prediction [21]. This method has also been extended to predict milling stability and surface location errors in subsequent studies [22].

Derived from the TFEA framework, the NIM calculates system dynamic responses via integral formulations. Ding et al. first developed the NIM using the Newton–Cotes and Gauss quadrature algorithms [23]. Zhang et al. applied this method to milling stability analysis considering modal coupling and regenerative effects [24], and further proposed a Simpson-integration-based NIM that constructs stability lobes by discretizing the milling cycle and employing Lagrange interpolation [25]. Dong et al. improved the NIM via Hermite-type integration, which delivers faster convergence and higher precision than conventional SDMs and FDMs [26]. Li et al. refined the NIM using the Newton–Cotes formula and validated its effectiveness through experimental tests [27]. Xia et al. established first- to fourth-order NIMs based on Lagrange integration. Their results revealed that although higher interpolation order improves prediction accuracy, it increases computational overhead, and the third-order NIM achieves the optimal balance between accuracy and efficiency [28]. Wang et al. constructed a hybrid interpolation scheme combining the Simpson formula and Hermite polynomials. Numerical validations demonstrate its excellent computational accuracy and efficiency, and a quantitative evaluation method for prediction precision based on mean square error was also proposed [29]. Recent studies have adopted multi-step numerical formulations to further enhance the modeling fidelity of state terms [30,31].

Among the aforementioned stability prediction methods, FDMs have attracted considerable attention due to their excellent prediction accuracy. Although FDM variants from first to fifth order have been developed using diverse interpolation polynomials, including Lagrange, Newton, and Hermite polynomials, systematic investigations of high-order FDM schemes remain inadequate. The interpolation order inherently determines the prediction accuracy and computational efficiency of FDMs. However, few studies have systematically explored the corresponding trade-off characteristics. To address this research gap, this study quantitatively analyzes the effects of Lagrange interpolation order, clarifies the performance rules of different interpolation orders, and determines the optimal FDM configuration.

## 2. Milling Dynamics Modeling

In milling operations, tool vibration frequently induces chatter marks on the machined workpiece surface. The interference between the tool flank and surface chatter marks forms an intrusion zone that triggers the plowing effect, namely process damping. Existing studies have demonstrated that the radial plowing force on the tool flank is proportional to the volume of extruded material, which can be formulated as [32]:

(1) $$F_{pr}=g(\varphi_{j})K_{sp}a_{p}S$$

where *K*_sp_ denotes the indentation coefficient, *a*_p_ represents the axial depth of cut, *S* is the cross-sectional area of the extruded material.

Process damping can be equivalently modeled as viscous damping and further simplified as:

(2) $$F_{pr}=C_{eq}\dot{r}(t)$$

where *C_eq_* represents the equivalent damping coefficient.

(3) $$C_{eq}=K_{sp}a_{p}W^{2}/\left(4v\right)$$

where *W* denotes the wear width of the flank face and v represents the cutting velocity, which can be expressed as:

(4) v=πDn/60

where *n* represents the spindle speed.

Based on friction theory, the tangential plowing force is formulated as:

(5) $$F_{pt}=\mu F_{pr}$$

where *μ* represents the friction coefficient.

Therefore, the process damping force can be expressed as:

(6) $$\left[\begin{array}{c} F_{pt}(t)\\ F_{pr}(t) \end{array}\right]=C_{eq}\left[\begin{array}{c} \mu \\ 1 \end{array}\right]\left[\begin{array}{cc} \sin\varphi_{j}(t) & \cos\varphi_{j}(t) \end{array}\right]\left[\begin{array}{c} \dot{x}(t)\\ \dot{y}(t) \end{array}\right]$$

Projecting the process damping force onto the X and Y directions of the milling system yields

(7) $$F_{p}=\left[\begin{array}{c} F_{px}(t)\\ F_{py}(t) \end{array}\right]=T_{i,j}(t)\left[\begin{array}{c} F_{pt}(t)\\ F_{pr}(t) \end{array}\right]$$

The process damping force is expanded as:

(8) $$F_{p}=\left[\begin{array}{c} F_{px}(t)\\ F_{py}(t) \end{array}\right]=-C_{eq}G\left[\begin{array}{c} \dot{x}(t)\\ \dot{y}(t) \end{array}\right]$$

where ***G*** represents the process damping coefficient matrix, described as:

(9) $$G=\left[\begin{array}{cc} g_{xx} & g_{xy}\\ g_{yx} & g_{yy} \end{array}\right]$$

where *g_xx_*, *g_xy_*, *g_yx_*, and *g_yy_* are the process damping coefficients, defined as:

(10) $$\begin{array}{l} g_{xx}(t)=\sum_{i=1}^{N_{t}}g(\varphi_{i}(t))\sin\varphi_{i}(t)[\mu \cos\varphi_{i}(t)+\sin\varphi_{i}(t)]\\ g_{xy}(t)=\sum_{i=1}^{N_{t}}g(\varphi_{i}(t))\cos\varphi_{i}(t)[\mu \cos\varphi_{i}(t)+\sin\varphi_{i}(t)]\\ g_{yx}(t)=\sum_{i=1}^{N_{t}}g(\varphi_{i}(t))\sin\varphi_{i}(t)[\cos\varphi_{i}(t)-\mu sin\varphi_{i}(t)]\\ g_{yy}(t)=\sum_{i=1}^{N_{t}}g(\varphi_{i}(t))\cos\varphi_{i}(t)[\cos\varphi_{i}(t)-\mu \sin\varphi_{i}(t)] \end{array}$$

Therefore, a two-degree-of-freedom milling dynamics equation is established, considering regenerative effects, modal coupling, and process damping, yielding:

(11) $$\left[\begin{array}{cc} m_{xx} & m_{xy}\\ m_{yx} & m_{yy} \end{array}\right]\left[\begin{array}{c} \ddot{x}(t)\\ \ddot{y}(t) \end{array}\right]+\left[\begin{array}{cc} c_{xx} & c_{xy}\\ c_{yx} & c_{yy} \end{array}\right]\left[\begin{array}{c} \dot{x}(t)\\ \dot{y}(t) \end{array}\right]+\left[\begin{array}{cc} k_{xx} & k_{xy}\\ k_{yx} & k_{yy} \end{array}\right]\left[\begin{array}{c} x(t)\\ y(t) \end{array}\right]=\left[\begin{array}{c} F_{x}(t)\\ F_{y}(t) \end{array}\right]+\left[\begin{array}{c} F_{px}(t)\\ F_{py}(t) \end{array}\right]$$

Let

(12) $$M_{1}=\left[\begin{array}{cc} m_{xx} & m_{xy}\\ m_{yx} & m_{yy} \end{array}\right]\;\;\;\;C_{1}=\left[\begin{array}{cc} c_{xx} & c_{xy}\\ c_{yx} & c_{yy} \end{array}\right]\;\;\;\;K_{1}=\left[\begin{array}{cc} k_{xx} & k_{xy}\\ k_{yx} & k_{yy} \end{array}\right]$$

Substitute Equations (8) and (12) into Equation (11):

(13) $$M_{1}\ddot{q}(t)+C_{1}\dot{q}(t)+K_{1}q(t)=a_{p}K_{c}[q(t)-q(t-T)]+C_{eq}G\dot{q}(t)$$

Equation (11) can be rearranged as:

(14) $$M_{1}\ddot{q}(t)+C_{c}\dot{q}(t)+K_{1}q(t)=a_{p}K_{c}[q(t)-q(t-T)]$$

where the matrix ***C_C_*** is described as:

(15) $$C_{c}=C_{1}-C_{eq}G$$

For the dynamic governing equation of the multi-factor coupled milling system, the direct integration strategy [9] is adopted for the numerical solution. The state vector ***x***(*t*) and periodic coefficient matrix ***p***(*t*) are subsequently introduced.

(16) $$\begin{array}{l} x(t)={\left[\begin{array}{cc} q(t) & p(t) \end{array}\right]}^{T}\\ p(t)=M_{1}\dot{q}(t)+C_{c}q(t)/2 \end{array}$$

Equation (14) can thus be simplified as:

(17) $$\dot{x}(t)=A_{0}x(t)+a_{p}B(t)\left[x(t)-x(t-T)\right]$$

where the matrix ***A*_0_** is expressed as:

(18) $$A_{0}=\left[\begin{array}{cc} -M_{1}^{-1}C_{c}/2 & M_{1}^{-1}\\ C_{c}M_{1}^{-1}C_{c}/4-K_{1} & -C_{c}M_{1}^{-1}/2 \end{array}\right]$$

Thus, the response of the milling system considering multi-factor influences can be described by Equation (17) as follows:

(19) $$x_{k+1}=e^{A_{0}\tau }x(k\tau )+a_{p}\int_{k\tau }^{t}\left\{e^{A_{0}(t-\xi )}B(k\tau +\tau -\xi )\left[x(k\tau +\tau -\xi )+x(k\tau +\tau -\xi -T)\right]\right\}d\xi$$

where ***B***(*kτ* + *τ* − *ξ*) represents the periodic coefficient term, ***x***(*kτ* + *τ* − *ξ*) denotes the system state term, and ***x***(*kτ* + *τ* − *ξ* − *T*) corresponds to the time-delay term.

## 3. Research on an Improved Full-Discretization Method

Based on the single-factor control variable method, the interpolation schemes for Equation (18) are designed as follows. First, the state term is interpolated via first- to fourth-order Lagrange polynomials, while the time-delay and periodic coefficient terms adopt linear interpolation. Second, the time-delay term is interpolated with first- to fourth-order Lagrange polynomials, while the state and periodic coefficient terms use linear interpolation. Third, the periodic coefficient term is interpolated by first- to fourth-order Lagrange polynomials, with linear interpolation applied to the state and time-delay terms. The detailed configurations of the above interpolation strategies are summarized in Table 1.

### 3.1. Analysis of Interpolation Schemes Based on the Single-Factor Variation Method

Taking the variable-order interpolation construction of the state-term FDM as an example, the interpolation schemes for the delay term and periodic coefficient term can be derived similarly. First, for the 111-FDM, linear interpolation is adopted for all state, time-delay, and periodic coefficient terms according to the single-factor variation approach. For the 211-FDM, the state term is interpolated using second-order Lagrange polynomials, while the time-delay and periodic coefficient terms are treated with linear interpolation. The 111 FDM and 211 FDM correspond to the first-order FDM [10] and second-order FDM [11] proposed in previous studies. The detailed derivation procedure is elaborated for the 311-FDM, and other methods follow the same analogy. For the 311 FDM, the periodic coefficient term is assumed to vary linearly with time over the interval [*kτ*,(*k* + 1)*τ*].

(20) $$B(k\tau +\tau -\xi )=B_{0}^{\left(k\right)}+B_{1}^{\left(k\right)}\xi$$

(21) $$\begin{array}{l} B_{0}^{\left(k\right)}=B_{k+1}\\ B_{1}^{\left(k\right)}=\left({\left(B\right)}_{k}-B_{k+1}\right)/\tau \end{array}$$

where the matrix ***B****_k_* denotes the value at time *t* = *kτ*, and ***B****_k+_*_1_ represents the value at time *t* = (*k* + 1)*τ*.

For the time-delay term:

(22) $$x(k\tau +\tau -\xi -T)=a_{1}x_{k-m}+b_{1}x_{k-m+1}$$

where the coefficients *a*_1_ and *b*_1_ are respectively given by:

(23) $$\begin{array}{l} a_{1}=\frac{\xi }{\tau }\\ b_{1}=1-\frac{\xi }{\tau } \end{array}$$

For the state term, interpolation is performed using a third-order Lagrange polynomial, selecting four nodal values: ***x****_k_*_−2_, ***x****_k_*_−1_, ***x****_k_* and ***x****_k_*_+1_

(24) $$x(k\tau +\tau -\xi )=a_{2}x_{k-2}+b_{2}x_{k-1}+c_{2}x_{k}+d_{2}x_{k+1}$$

where the coefficients *a*_2_, *b*_2_, *c*_2_, and *d*_2_ are respectively given by:

(25) $$\begin{array}{l} a_{2}=\frac{\xi }{3\tau }-\frac{\xi^{2}}{2\tau^{2}}+\frac{\xi^{3}}{6\tau^{3}}\\ b_{2}=-\frac{3\xi }{2\tau }+\frac{2\xi^{2}}{\tau^{2}}-\frac{\xi^{3}}{2\tau^{3}}\\ c_{2}=\frac{3\xi }{\tau }-\frac{5\xi^{2}}{2\tau^{2}}+\frac{\xi^{3}}{2\tau^{3}}\\ d_{2}=1-\frac{11\xi }{6\tau }+\frac{\xi^{2}}{\tau^{2}}-\frac{\xi^{3}}{6\tau^{3}} \end{array}$$

Substituting Equations (20), (22) and (24) into Equation (19), Equation (19) can be reformulated as:

(26) $$x_{k+1}=F_{k1}x_{k+1}+(F_{0}+F_{k})x_{k}+F_{kp1}x_{k-1}+F_{kp2}x_{k-2}-F_{1m}x_{k+1-m}-F_{m}x_{k-m}$$

where the matrices ***F**_k_*_1_, ***F****_k_*, ***F**_kp_*_1_, ***F****_kp_*_2_, ***F***_1*m*_, ***F**_m_*, and ***F***_0_ are specifically defined as:

(27) $$\begin{array}{l} F_{k1}=(\Phi_{1}-\frac{11\Phi_{2}}{6\tau }+\frac{\Phi_{3}}{\tau^{2}}-\frac{\Phi_{4}}{6\tau^{3}})B_{0}^{\left(k\right)}+(\Phi_{2}-\frac{11\Phi_{3}}{6\tau }+\frac{\Phi_{4}}{\tau^{2}}-\frac{\Phi_{5}}{6\tau^{3}})B_{1}^{\left(k\right)}\\ F_{k}=(\frac{3\Phi_{2}}{\tau }-\frac{5\Phi_{3}}{2\tau^{2}}+\frac{\Phi_{4}}{2\tau^{3}})B_{0}^{\left(k\right)}+(\frac{3\Phi_{3}}{\tau }-\frac{5\Phi_{4}}{2\tau^{2}}+\frac{\Phi_{5}}{2\tau^{3}})B_{1}^{\left(k\right)}\\ F_{kp1}=(-\frac{3\Phi_{2}}{2\tau }+\frac{2\Phi_{3}}{\tau^{2}}-\frac{\Phi_{4}}{2\tau^{3}})B_{0}^{\left(k\right)}+(-\frac{3\Phi_{3}}{2\tau }+\frac{2\Phi_{4}}{\tau^{2}}-\frac{\Phi_{5}}{2\tau^{3}})B_{1}^{\left(k\right)}\\ F_{kp2}=(\frac{\Phi_{2}}{3\tau }-\frac{\Phi_{3}}{2\tau^{2}}+\frac{\Phi_{4}}{6\tau^{3}})B_{0}^{\left(k\right)}+(\frac{\Phi_{3}}{3\tau }\Phi_{3}-\frac{\Phi_{4}}{2\tau^{2}}+\frac{\Phi_{5}}{6\tau^{3}})B_{1}^{\left(k\right)}\\ F_{1m}=(\Phi_{1}-\frac{\Phi_{2}}{\tau })B_{0}^{\left(k\right)}+(\Phi_{2}-\frac{\Phi_{3}}{\tau })B_{1}^{\left(k\right)}\\ F_{m}=\frac{\Phi_{2}}{\tau }B_{0}^{\left(k\right)}+\frac{\Phi_{3}}{\tau }B_{1}^{\left(k\right)}\\ F_{0}=\Phi_{0} \end{array}$$

where ***Φ*****_0_**, ***Φ*_1_**, ***Φ*_2_**, ***Φ*_3_**, ***Φ*_4_** and ***Φ*_5_** are described as:

(28) $$\begin{array}{l} \Phi_{0}=e^{A_{0}\tau }\\ \Phi_{1}=\int_{0}^{\tau }e^{A_{0}\xi }d\xi =A_{0}^{-1}(\Phi_{0}-I)\\ \Phi_{2}=\int_{0}^{\tau }\xi e^{A_{0}\xi }d\xi =A_{0}^{-1}(\tau \Phi_{0}-\Phi_{1})\\ \Phi_{3}=\int_{0}^{\tau }\xi^{2}e^{A_{0}\xi }d\xi =A_{0}^{-1}(\tau^{2}\Phi_{0}-2\Phi_{2})\\ \Phi_{4}=\int_{0}^{\tau }\xi^{3}e^{A_{0}\xi }d\xi =A_{0}^{-1}(\tau^{3}\Phi_{0}-3\Phi_{3})\\ \Phi_{5}=\int_{0}^{\tau }\xi^{4}e^{A_{0}\xi }d\xi =A_{0}^{-1}(\tau^{4}\Phi_{0}-4\Phi_{4}) \end{array}$$

When the matrix (***I*** − ***F****_k_*_1_) is non-singular, Equation (26) simplifies to:

(29) $$x_{k+1}=G_{11}x_{k}+G_{p1}x_{k-1}+G_{p2}x_{k-2}-G_{1m}x_{k+1-m}-G_{m}x_{k-m}$$

where the matrices ***G***_11_, ***G**_p_*_1_, ***G**_p_*_2_, ***G***_1*m*_ and ***G**_m_* are respectively given by:

(30) $$\begin{array}{l} G_{11}={\left(I-F_{k1}\right)}^{-1}(F_{0}+F_{k})\\ G_{p1}={\left(I-F_{k1}\right)}^{-1}F_{kp1}\\ G_{p2}={\left(I-F_{k1}\right)}^{-1}F_{kp2}\\ G_{1m}={\left(I-F_{k1}\right)}^{-1}F_{1m}\\ G_{m}={\left(I-F_{k1}\right)}^{-1}F_{m} \end{array}$$

When the inverse matrix (***I*** − ***F****_k_*_1_)^−1^ exists, a discrete mapping relationship is constructed, leading to Equation (31):

(31) $$y_{k+1}=D_{k}y_{k}$$

where the vector ***y**_k__+_*_1_ is given by:

(32) $$y_{k+1}=col(x_{k},x_{k-1},\cdots ,x_{k+1-m},x_{k-m})$$

where the vector ***D**_k_* is given by:

(33) $$D_{k}=\left[\begin{array}{ccccccc} G_{11} & G_{p1} & G_{p2} & \ldots & 0 & G_{1m} & G_{m}\\ I & 0 & 0 & \ldots & 0 & 0 & 0\\ 0 & I & 0 & \ldots & 0 & 0 & 0\\ 0 & 0 & I & \ldots & 0 & 0 & 0\\ \vdots & \vdots & \vdots & \ddots & \vdots & \vdots & \vdots \\ 0 & 0 & 0 & \ldots & I & 0 & 0\\ 0 & 0 & 0 & \ldots & 0 & I & 0 \end{array}\right]$$

where the matrix ***D_k_*** is a series matrix representing the state transition matrix. Over one milling period, the state transition matrix ***Φ*** is expressed as:

(34) $$\Phi =D_{m-1}D_{m-2}\cdots D_{1}D_{0}$$

Subsequently, the vector ***y****_m_* is obtained:

(35) $$y_{m}=\Phi y_{0}$$

Then, according to Floquet theory [33], the stability of the system is determined. The system is stable when the magnitudes of all eigenvalues of the matrix ***Φ*** are less than 1. Conversely, the system is in a state of chatter.

### 3.2. Analysis of the Influence of Interpolation Order on Stability Prediction

To comprehensively evaluate the stability prediction performance, the aforementioned FDMs are utilized to predict the stability of a single-degree-of-freedom milling system. The effects of interpolation orders for state, time-delay, and periodic coefficient terms on the predictive performance of FDMs are systematically analyzed. A classic single-degree-of-freedom milling system, which is widely adopted in stability prediction research, is selected for parametric analysis. The detailed parameters of the system are listed in Table 2.

#### 3.2.1. Analysis of the Influence of Interpolation Order on Convergence Rate

Reference [6] introduces the local discretization error to characterize the convergence of stability prediction methods. Let the exact value |μ_0_| of the critical eigenvalue of the state transition matrix, and let its estimated value be |μ|. The difference between them defines the local discretization error. Here, the number of discrete periods is a function of the local discretization error. When this number is sufficiently large, the local discretization error approaches zero [25]. The exact value is taken as the estimated value obtained by the second-order FDM [11] with m = 200.

**Figure 2:**
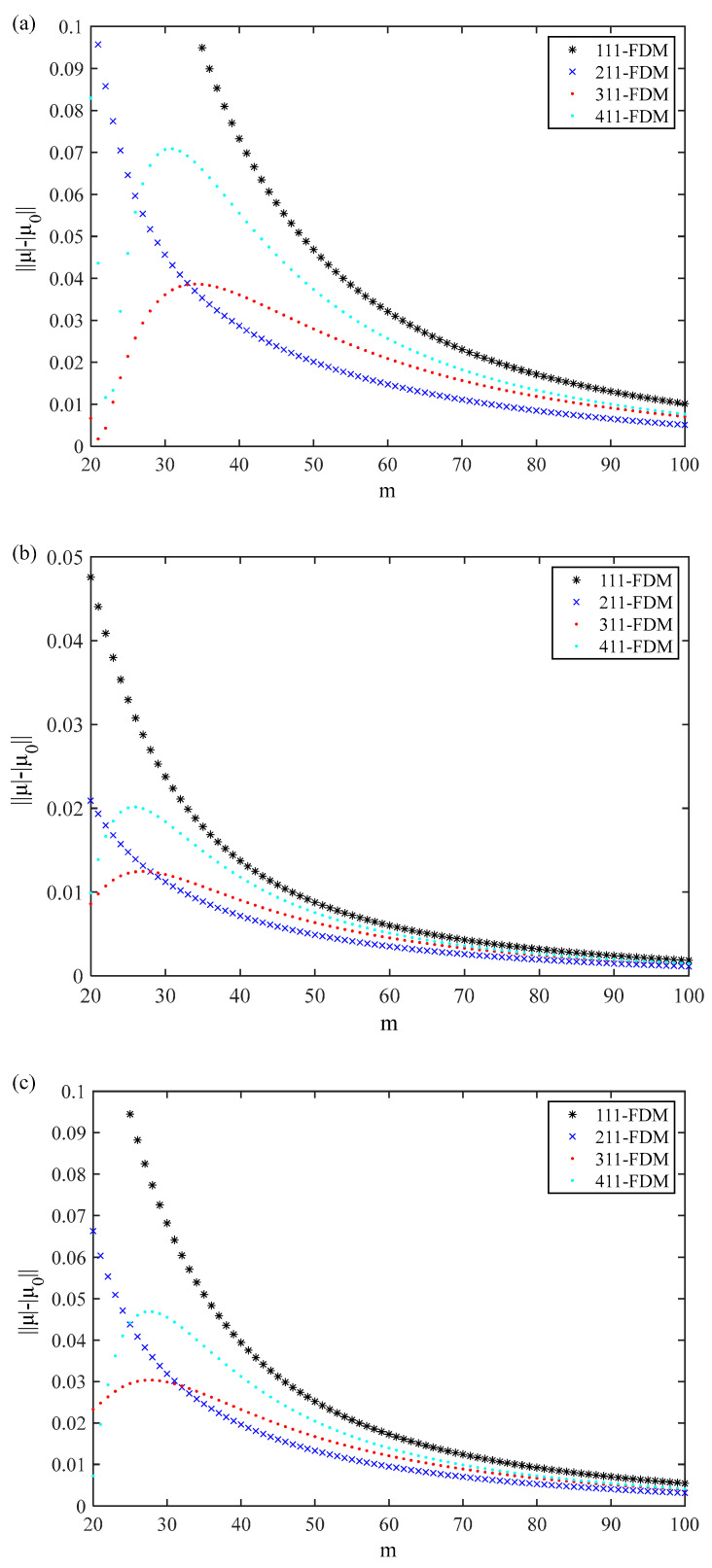
Convergences of the eigenvalues for the four FDMs: (**a**) *a*_p_ = 0.2; (**b**) *a*_p_ = 0.5; (**c**) *a*_p_ = 1.

**Figure 3:**
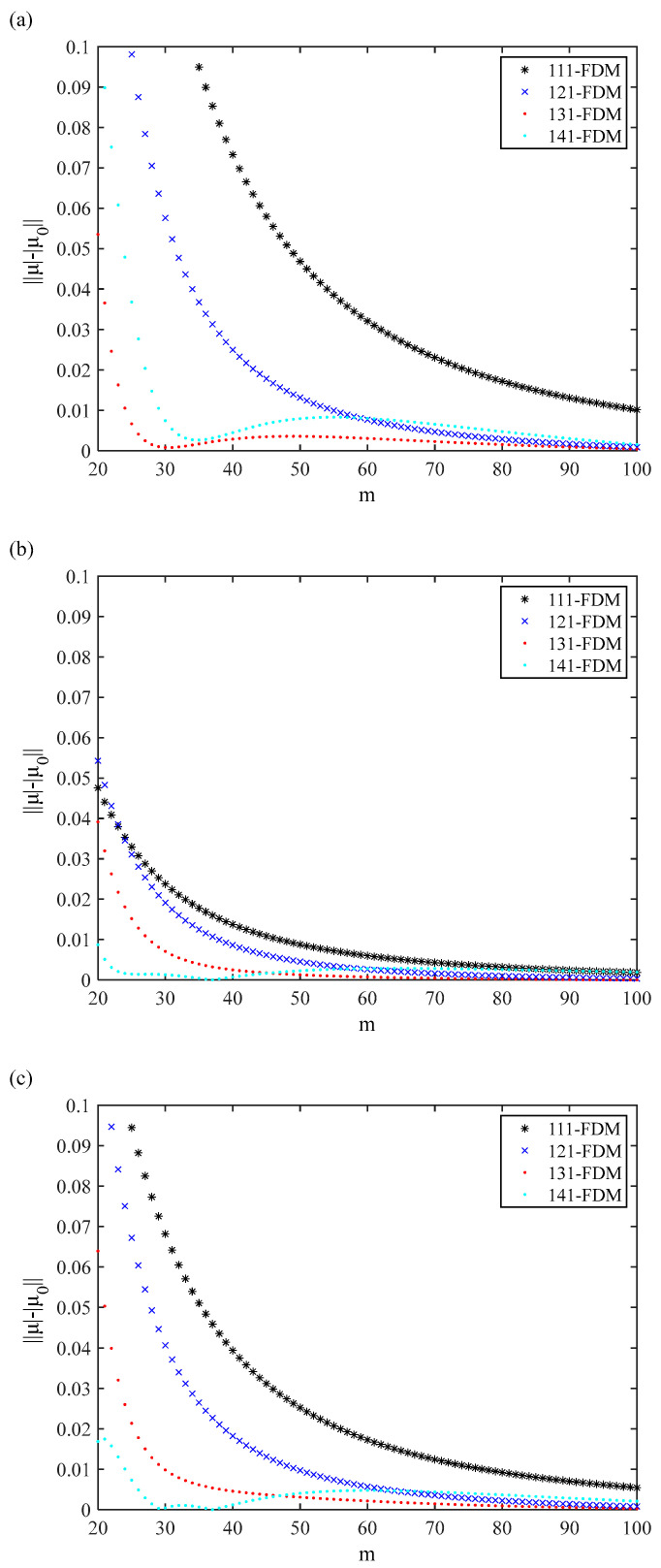
Convergences of the eigenvalues for the four FDMs: (**a**) *a*_p_ = 0.2; (**b**) *a*_p_ = 0.5; (**c**) *a*_p_ = 1.

**Figure 4:**
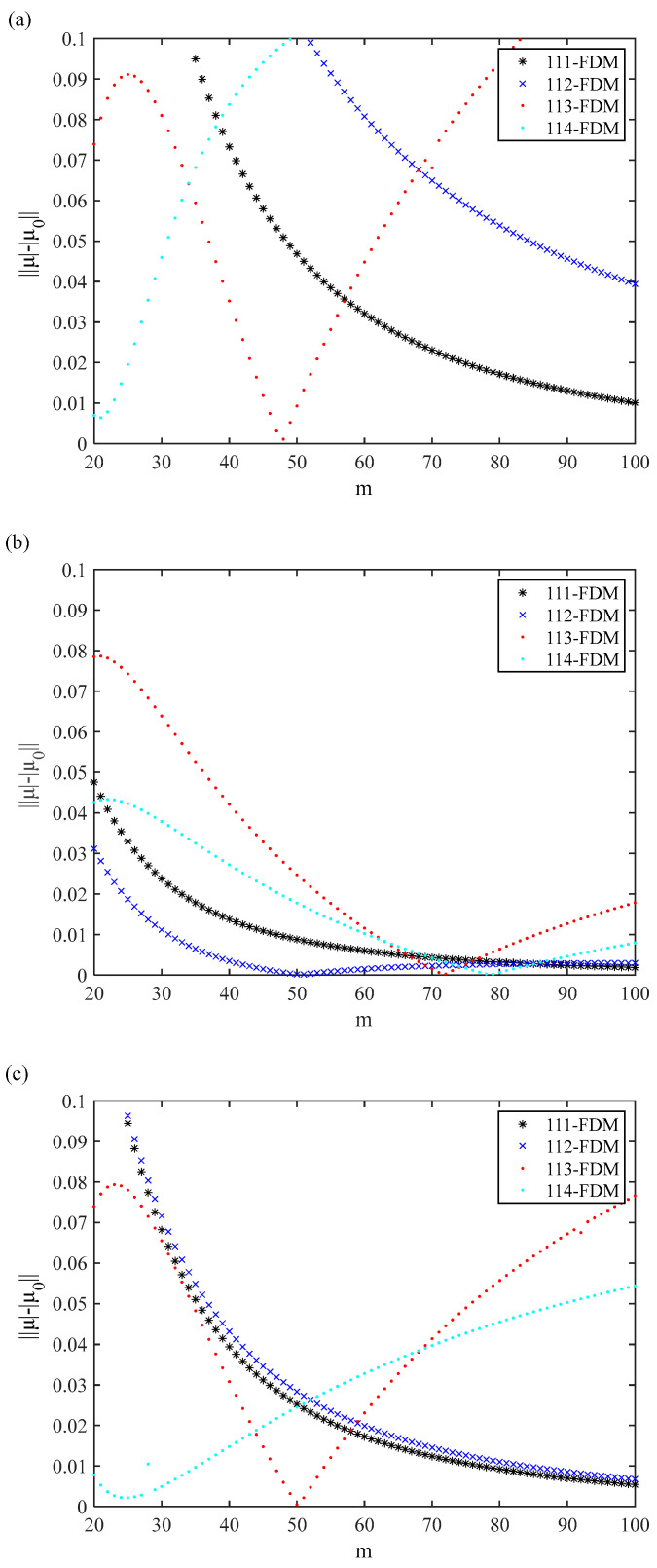
Convergences of the eigenvalues for the four FDMs: (**a**) *a*_p_ = 0.2; (**b**) *a*_p_ = 0.5; (**c**) *a*_p_ = 1.

The main cutting parameters adopted for the single-DOF milling system are as follows: spindle speed of 5000 rpm, down milling, radial depth of cut ratio of 1, and axial depths of cut of 0.2 mm, 0.5 mm, and 1 mm. The remaining parameters are listed in Table 2. Convergence rate curves are calculated under different full-discretization schemes. Figure 2 shows the results of 111, 211, 311 and 411 FDMs. Figure 3 presents the results of 111, 121, 131 and 141 FDMs, while Figure 4 illustrates the results of 111, 112, 113 and 114 FDMs.

From Figure 2, it can be observed that the local discretization errors of the four FDMs gradually decrease with the increase in discretization number, which verifies the effectiveness of the full-discretization method. Comparative analysis of convergence curves shows that increasing the state-term interpolation order from the first to the second order accelerates the convergence rate of the 211 FDM compared with the 111 FDM. However, further increasing the interpolation order to the third and fourth orders reduces the convergence performance. The 311 FDM exhibits a slower convergence rate than the 211 FDM, while the 411 FDM performs even worse than the 311 FDM. These results indicate that simply adopting higher-order polynomials for state-term interpolation cannot improve the computational efficiency of FDMs.

Taking Figure 2a as an example, when the discretization number is set to m = 50, the local discretization errors of the 111 FDM, 211 FDM, 311 FDM, and 411 FDM are 0.047, 0.020, 0.028, and 0.037, respectively. This demonstrates that, under the same discretization number, the 211 FDM yields the critical eigenvalue of the state transition matrix closest to the exact value and achieves the optimal prediction accuracy.

From Figure 3, it can be observed that raising the interpolation order of Lagrange polynomials for the time-delay term from the first to the third order accelerates the convergence rate of the corresponding FDMs. When the order is further increased to the fourth order, however, the convergence rate of the 141 FDM declines rather than improves. For m ≤ 46, the 141 FDM maintains a faster convergence rate than the other three methods. For m > 46, its convergence becomes slower than that of the 131 FDM, as presented in Figure 3a,b. In Figure 3c, the 141 FDM always converges more slowly than the 131 FDM. The curves reveal that the 141 FDM shows obvious performance fluctuation, while the 131 FDM achieves more stable and higher prediction efficiency.

In Figure 4, as m rises from 20 to 100, the convergence curve of the 113 FDM declines initially and then rises, which means this method may fail to converge and thus has poor reliability for stability prediction. Likewise, the curve of the 114 FDM does not decrease with the growth of m, making it inappropriate for prediction. By comparison, Figure 4a shows that the 112 FDM converges better than the 111 FDM. In Figure 4b,c, the 111 FDM presents superior convergence, and the performance gap between the two methods gradually enlarges. Consequently, increasing the interpolation order of the periodic coefficient term cannot effectively improve the convergence of FDMs. Linear interpolation for the periodic term delivers the best convergence performance.

#### 3.2.2. Analysis of the Influence of Interpolation Order on SLDs

**Figure 5:**
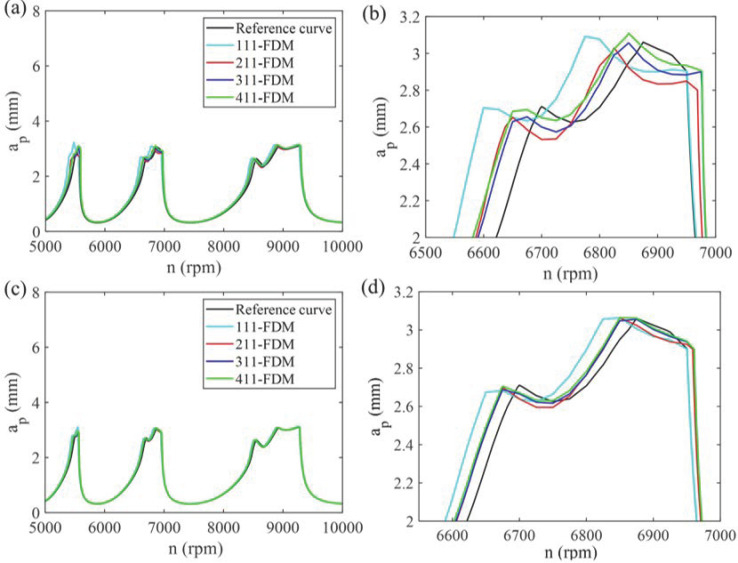
SLDs and local magnification diagrams for the four FDMs: (**a**) global view and (**b**) local magnification for m = 40; (**c**) global view and (**d**) local magnification for m = 60.

**Figure 6:**
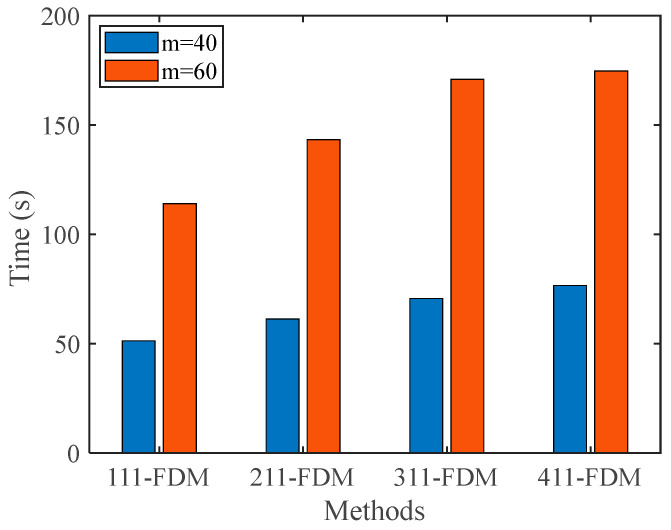
Computational time for the four FDMs.

**Figure 7:**
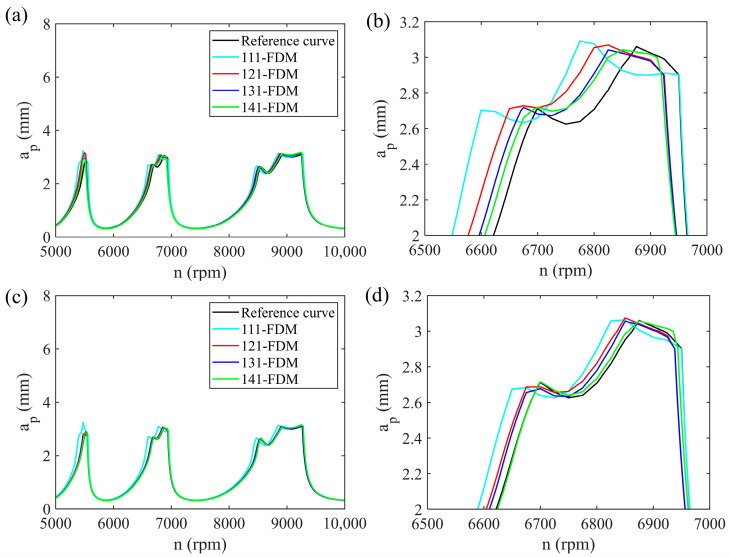
SLDs and local magnification diagrams for the four FDMs: (**a**) global view and (**b**) local magnification for m=40; (**c**) global view and (**d**) local magnification for m = 60.

**Figure 8:**
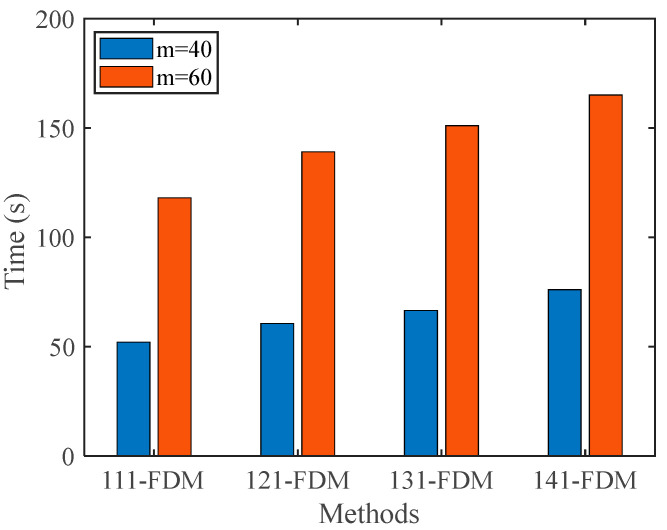
Computational time for the four FDMs.

**Figure 9:**
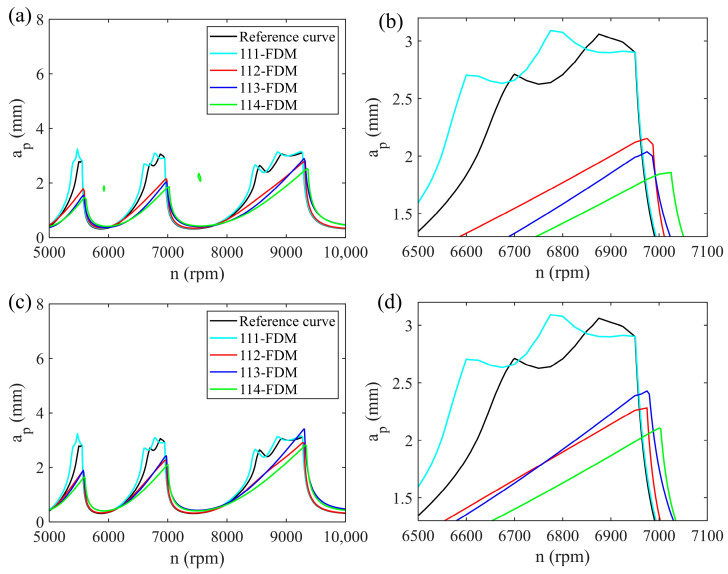
SLDs and local magnification diagrams for the four FDMs: (**a**) global view and (**b**) local magnification for m=40; (**c**) global view and (**d**) local magnification for m = 60.

**Figure 10:**
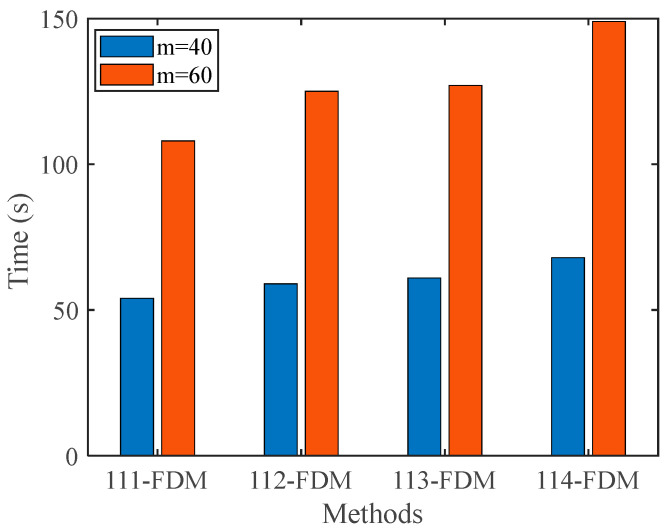
Computational time for the four FDMs.

To compare the prediction performance of different methods, we calculate the stability lobe diagrams (SLDs) and evaluate their prediction accuracy and computational time. The SLDs are obtained at m = 40 and 60. Figure 5 presents the results of the 111 FDM, 211 FDM, 311 FDM and 411 FDM, and the corresponding computational time is shown in Figure 6. Figure 7 displays the results of the 111 FDM, 121 FDM, 131 FDM and 141 FDM, and the relevant computational time is given in Figure 8. Figure 9 shows the results of the 111 FDM, 112 FDM, 113 FDM and 114 FDM, and the corresponding computational time is provided in Figure 10.

Figure 5a,c illustrate the calculated SLDs, where locally magnified views of the boxed regions are provided for detailed comparison. The black curve denotes the reference SLD, which is calculated via the second-order FDM with m = 200. Compared with the 111 FDM and 411 FDM, the SLDs predicted by the 211 FDM and 311 FDM match better with the reference curve, demonstrating higher computational accuracy. From Figure 6, the computational time for stability lobe prediction increases continuously with the growth of the state-term interpolation order. Considering both accuracy and efficiency, the 211 FDM achieves the optimal overall prediction performance, followed by the 311 FDM. Although the 111 FDM provides lower prediction accuracy than the 411 FDM, it possesses a distinctly higher computational efficiency.

When m = 40, as shown in Figure 7a,b, the SLD obtained from the 141 FDM is closer to the reference curve, which is consistent with the previous convergence results and indicates superior computational accuracy of the 141 FDM at this discretization level. In contrast, the 131 FDM produces SLD curves that better fit the reference results at m = 60. In terms of computational efficiency, Figure 8 reveals that the computational time increases with the interpolation order of the time-delay term. Accordingly, the 131 FDM achieves the optimal comprehensive prediction performance among the four compared methods.

From Figure 9a,c, the stability lobe diagram calculated by the 111-FDM shows the best agreement with the reference curve. In comparison, the SLDs obtained from the 112-FDM, 113-FDM, and 114-FDM present obvious deviations from the reference curve, which is consistent with the aforementioned convergence results. According to the computational time shown in Figure 10, higher interpolation orders for the periodic coefficient term correspond to longer computation time. Overall, the FDMs with high-order interpolation for periodic coefficient terms (112-FDM, 113-FDM, and 114-FDM) fail to improve the stability prediction performance and produce considerable prediction errors instead. Therefore, higher-order interpolation of the periodic coefficient term negatively affects the overall prediction accuracy. For FDM construction, linear interpolation of the periodic coefficient term achieves the optimal prediction performance.

## 4. Optimization of the Order of the Improved Full-Discretization Method

Based on the above analysis regarding the effects of interpolation orders of the system state term, time-delay term, and periodic coefficient term on FDM performance, the main conclusions are summarized as follows. The state term achieves the optimal prediction performance at the second-order interpolation, followed by the third-order. The time-delay term performs best with third-order interpolation, followed by second-order. For the periodic coefficient term, the first-order interpolation exhibits the highest accuracy. Accordingly, the recommended interpolation order combinations for the full-discretization method are listed in Table 3. The corresponding methods for Combinations XI to XIV are defined as 221-FDM, 231-FDM, 321-FDM, and 331-FDM, respectively.

### 4.1. Determination of the Optimal Interpolation Order for the Improved Full-Discretization Method

To further compare the stability prediction performance of the four proposed FDMs (221-FDM, 231-FDM, 321-FDM, and 331-FDM), the same single-degree-of-freedom milling system and parameter settings are adopted for numerical validation to analyze their convergence characteristics and predicted stability boundaries.

**Figure 11:**
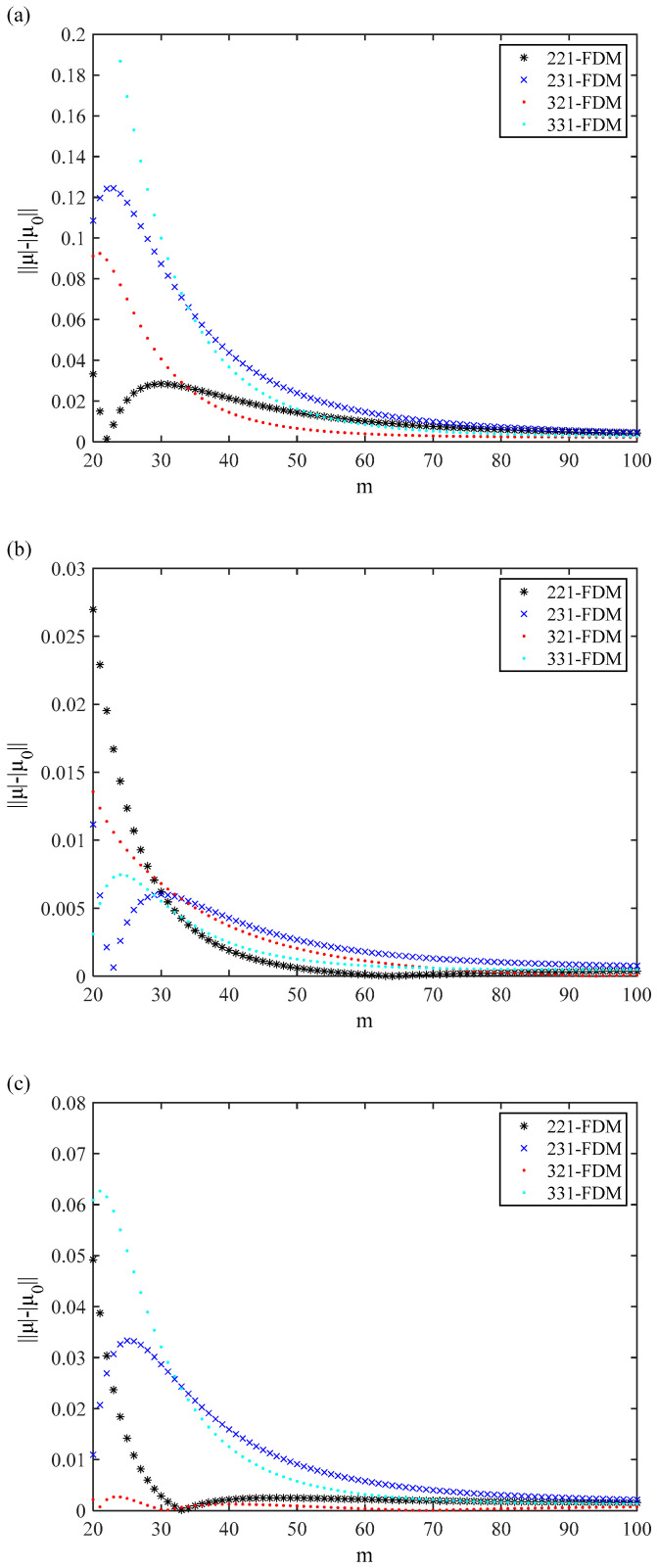
Convergences of the eigenvalues for the four FDMs: (**a**) ap = 0.2; (**b**) ap = 0.5; (**c**) ap = 1.

First, convergence analysis is performed with identical cutting conditions: a spindle speed of 5000 rpm, a radial depth of cut ratio of 1, and axial depths of cut of 0.2 mm, 0.5 mm, and 1 mm. The convergence rate curves of the 221 FDM, 231 FDM, 321 FDM, and 331 FDM under the three axial depth-of-cut conditions are calculated and presented in Figure 11.

From Figure 11, it can be observed that among the four FDMs, the 231 FDM exhibits the slowest convergence, followed by the 331 FDM. For the 221 FDM and 321 FDM, the 221 FDM converges faster when m < 80, while the 321 FDM achieves better convergence performance when m > 80. As illustrated in Figure 11, the 321 FDM maintains a faster convergence rate than the 221 FDM. Overall, the 321 FDM presents the most stable and superior convergence performance.

**Figure 12:**
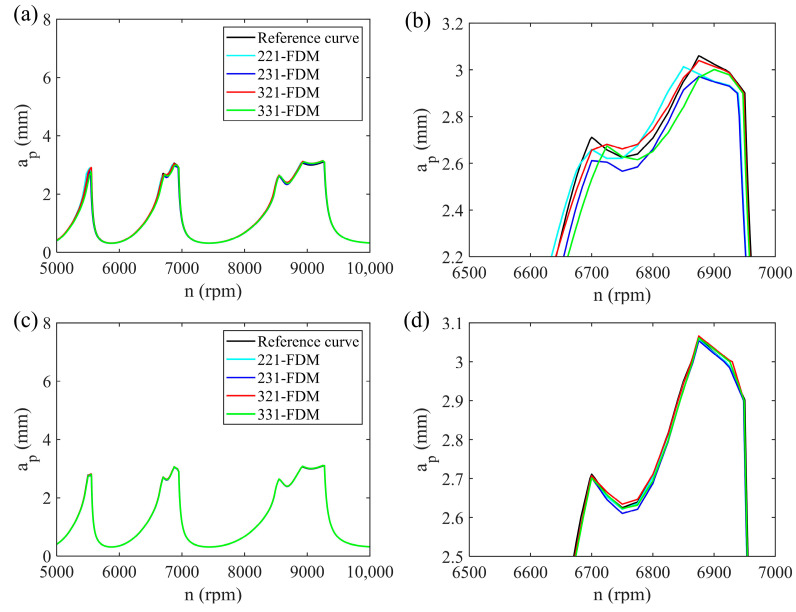
SLDs and local magnification diagrams for the four FDMs: (**a**) global view and (**b**) local magnification for m=40; (**c**) global view and (**d**) local magnification for m = 80.

To further compare the predictive performance of the 221 FDM, 231 FDM, 321 FDM, and 331 FDM, SLDs are calculated to evaluate their prediction accuracy and computational efficiency. The SLDs of the four FDMs are obtained at m = 40 and m = 80, as depicted in Figure 12. Figure 12a,c present the stability boundaries corresponding to spindle speeds ranging from 5000 rpm to 10,000 rpm and axial depths of cut from 0 to 8 mm. Figure 12b,d provide local magnification of a typical stability lobe to clearly compare curve distributions. In the enlarged view of Figure 12b, the SLD curve predicted by the 321 FDM shows the closest agreement with the reference curve. Consistent results can be observed in Figure 12d, further verifying that the 321 FDM possesses the highest prediction accuracy among the four methods.

**Figure 13:**
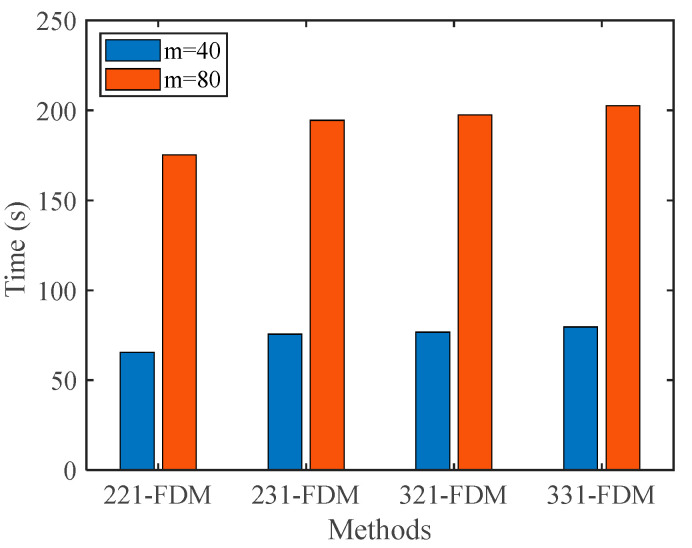
Computational time for the four FDMs.

In Figure 13, in terms of computational time, the 221 FDM consumes the shortest computation duration, while the 231 FDM, 321 FDM, and 331 FDM yield comparable computational time. This phenomenon is determined by the total interpolation order adopted for FDM construction. A higher combined interpolation order of the state, time-delay, and periodic coefficient terms results in greater computational cost, and vice versa.

Based on the above comprehensive analysis, the 321 FDM achieves the optimal overall prediction performance among the four developed methods (221 FDM, 231 FDM, 321 FDM, and 331 FDM). The previous sections identified the 211 FDM and 131 FDM as relatively effective methods through single-parameter analysis. Such performance discrepancies occur because the single-factor variation method adopted in the earlier analysis separately explores the influence of each individual interpolation order while ignoring the coupling effects of the other two terms. In comparison, this section optimizes the interpolation order configuration by fully considering the coupling interactions among the state, time-delay, and periodic coefficient terms. The analytical results confirm that the improved full-discretization 321 FDM possesses superior comprehensive prediction performance for milling stability.

### 4.2. Comparison with Existing Discretization Methods

The improved 321-FDM requires comparative validation against existing discretization methods to verify its performance enhancement. Previous analyses concerning interpolation order effects and parameter optimization have already included comparisons with several classic discretization methods, including the first-order 111-FDM [10], second-order 211-FDM [11] and 221-FDM [12], and fourth-order 141-FDM [18]. Accordingly, these comparative results are not repeated herein.

Therefore, the SDM [7] and a recently proposed third-order FDM [14] are selected for further benchmark comparison. The adopted third-order FDM (3H2L-FDM) utilizes third-order Hermite polynomials for the state term, second-order Lagrange polynomials for the time-delay term, and first-order Lagrange polynomials for the periodic coefficient term [14]. Its interpolation configuration is highly similar to the optimized 321-FDM proposed in this work, which ensures a reasonable and targeted comparison. To unify the naming convention with 3H2L-FDM, the 321-FDM is correspondingly renamed 3L2L-FDM.

**Figure 14:**
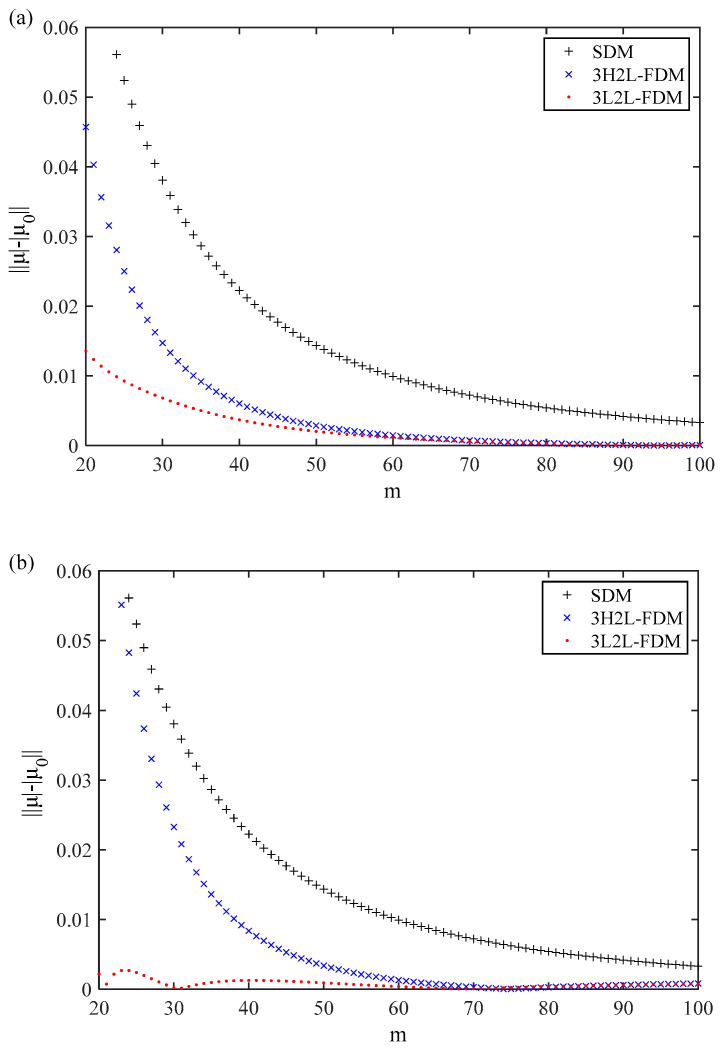
Convergences of the eigenvalues for the four FDMs: (**a**) ap = 0.2; (**b**) ap = 0.5.

Similarly, all calculations are performed on the single-degree-of-freedom milling system with the system parameters listed in Table 2. The adopted cutting parameters are set as follows: spindle speed of 5000 rpm, climb milling, radial depth of cut ratio of 1, and axial depths of cut of 0.2 mm and 0.5 mm. The convergence rate curves corresponding to axial depths of cut of 0.2 mm and 0.5 mm are calculated via the SDM, 3H2L FDM, and 3L2L FDM, as depicted in Figure 14. As presented in Figure 14b, when the discretization number is m = 40, the local discretization errors of the SDM, 3H2L FDM, and 3L2L FDM are 0.022, 0.008, and 0.001, respectively. The numerical results demonstrate that the improved 3L2L-FDM possesses the highest prediction accuracy under identical discretization parameters, followed by the 3H2L-FDM and SDM. As m increases from 20 to 100, the 3L2L-FDM exhibits a faster convergence rate than the 3H2L-FDM, while the SDM shows the slowest convergence performance.

**Figure 15:**
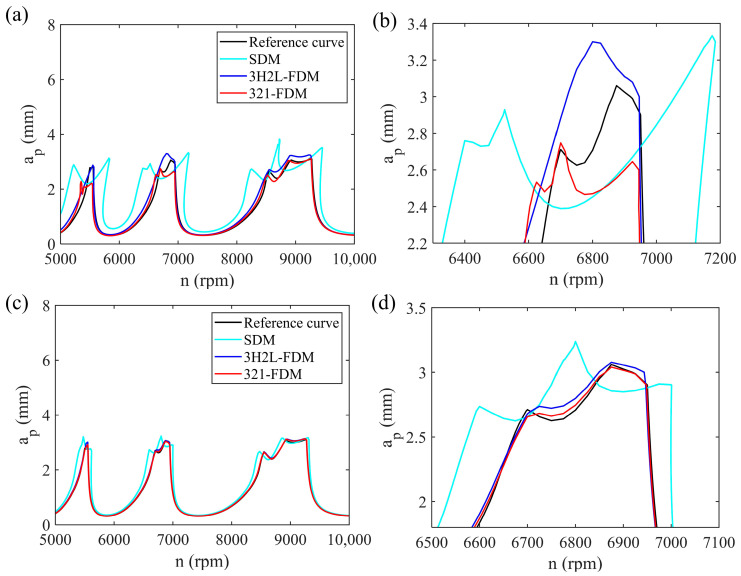
SLDs and local magnification diagrams for the four FDMs: (**a**) global view and (**b**) local magnification for m = 20; (**c**) global view and (**d**) local magnification for m = 40.

**Figure 16:**
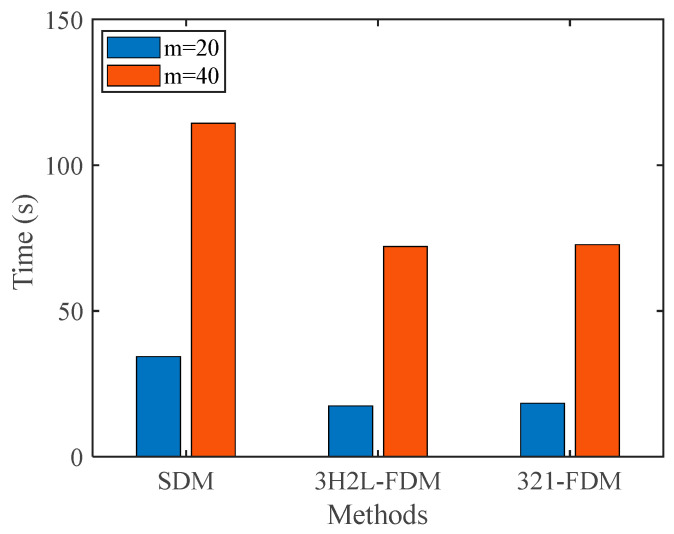
Computational time for three discretization methods.

Subsequently, stability prediction analysis is further carried out. The SLDs predicted at m = 20 and 40 are illustrated in Figure 15. The results show that the SLD curve obtained by the 3L2L FDM fits best with the reference curve, confirming its superior prediction accuracy. In terms of computational time, as presented in Figure 16, the 3L2L FDM and 3H2L FDM consume comparable computation time, both of which are less time-consuming than the SDM. Overall, among the SDM, 3H2L FDM and 3L2L FDM, the proposed 3L2L FDM delivers the optimal stability prediction performance in terms of both accuracy and efficiency.

## 5. Experimental Verification

Milling stability prediction experiments primarily consist of three parts: modal testing, milling force coefficient identification, and milling process verification. The detailed models of the experimental equipment are listed in Table 4.

### 5.1. Parameter Identification of the Cast Iron Milling System

As discussed above, stability prediction relies on the dynamic parameters of the machining system, cutting force coefficients, and a reliable prediction method. The improved prediction method has been elaborated in the previous sections. To realize accurate stability prediction for cast iron milling systems, it is necessary to acquire the dynamic parameters of the cutter-spindle system and the corresponding cutting force coefficients.

#### 5.1.1. Modal Testing

**Figure 17:**
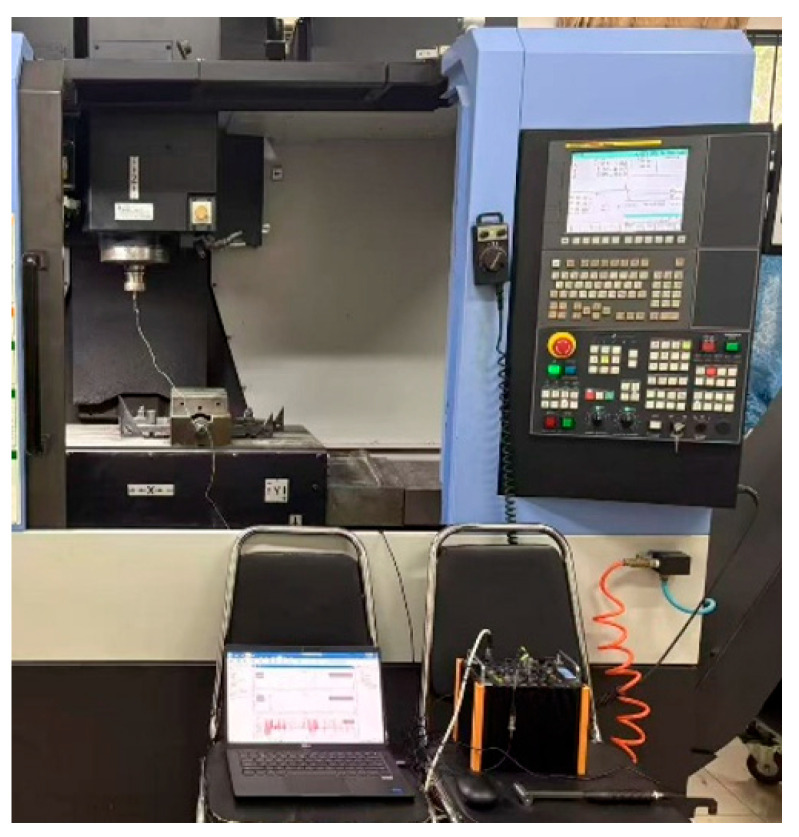
Photo of modal testing.

A hammer-impact modal test is performed to acquire the modal parameters of the machining system. The detailed testing procedure is described as follows. The milling tool is clamped on the machine tool spindle (Doosan Machine Tools (Yantai) Co., Ltd., Yantai, Shandong, China), and an accelerometer is installed at the tool tip, while an impact hammer is adopted to excite the tool end. The induced tool vibration responses are collected by the accelerometer, and the impact force signals are synchronously recorded by the impact hammer. Both vibration and force signals are transmitted to the signal acquisition module (Jiangsu Donghua Testing Technology Co., Ltd., Taizhou, Jiangsu, China). Subsequent signal analysis enables the identification of the modal parameters of the tool system. The on-site modal test setup is presented in Figure 17. The detailed specifications of the milling cutter are listed in Table 5, and the identified modal parameters of the cutter system are summarized in Table 6.

#### 5.1.2. Cutting Force Coefficient Identification

The rapid calibration method is widely used to determine milling force coefficients. In slot-milling tests, the entry and exit angles are fixed at 0° and 180°, respectively. The average milling forces in the X and Y directions can be expressed by the following equations [35]:

(36) $$\left\{\begin{array}{l} \bar{F_{x}}=\frac{N_{t}a_{p}}{4}K_{r}f_{z}+\frac{N_{t}a_{p}}{\pi }K_{re}\\ \bar{F_{y}}=\frac{N_{t}a_{p}}{4}K_{t}f_{z}+\frac{N_{t}a_{p}}{\pi }K_{te} \end{array}\right.$$

where *N_t_* is the number of tool teeth and *K_t_* and *K_r_* are the cutting force coefficients.

From Equation (36), the cutting force coefficients can be determined through milling experiments with varying feed rates. The cutter parameters adopted in the calibration tests are listed in Table 5, and the corresponding machining parameters are provided in Table 7. During the experiments, milling forces under each cutting condition are measured using a Kistler dynamometer (Kistler Group, Winterthur, Switzerland). Linear regression analysis is subsequently performed to obtain the cutting force coefficients, which are determined as Kt = 887 N/mm^2^ and Kr = 337 N/mm^2^.

### 5.2. Stability Prediction and Experimental Validation of the Cast Iron Milling System

**Figure 18:**
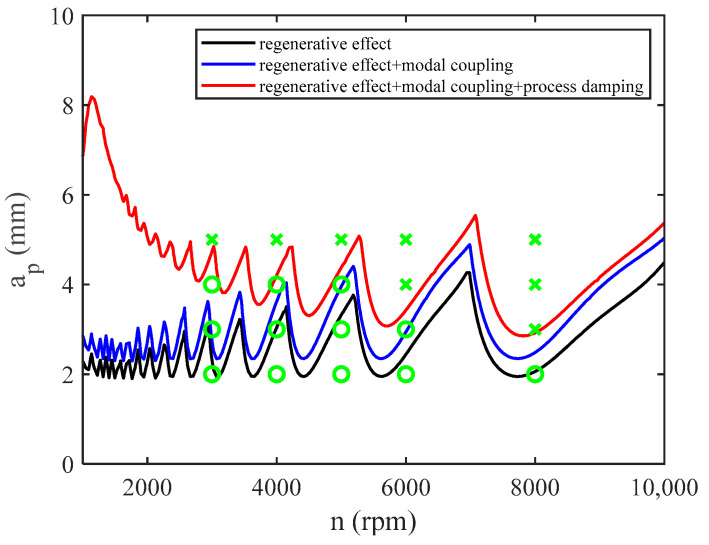
SLD and testing results of milling operations.

With the acquired modal parameters and cutting force coefficients, the improved method is adopted to predict the SLD of the cast-iron milling system, as illustrated in Figure 18.

**Figure 19:**
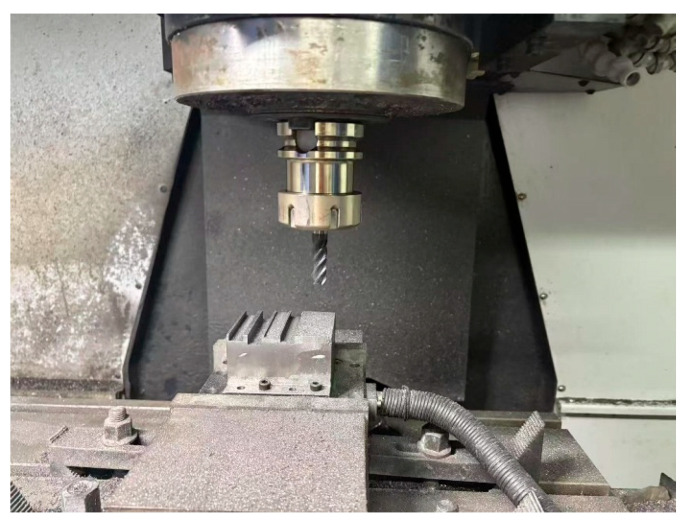
Milling processing.

To validate the accuracy of the predicted stability lobe diagram, machining parameters are selected according to the obtained SLD. A total of 20 groups of milling experiments are designed, covering four axial depths of cut and five spindle speeds, as summarized in Table 8. The milling experimental setup is displayed in Figure 19. During the tests, milling force signals under each cutting condition are collected, and the machined surface morphology is observed to judge the system stability corresponding to each parameter group. The experimental results (stable or chatter states) are finally marked in Figure 18.

In Figure 18, three types of SLD curves are plotted, each considering different dynamic factors: only the regenerative effect, the combination of regenerative effect and modal coupling, and the coupling of regenerative effect, modal coupling, and process damping. In the figure, the circle symbol “○” represents stable cutting conditions, while the cross symbol “×” denotes chatter conditions. The experimental data match best with the SLD curve predicted by the multi-factor dynamic model that integrates regenerative effect, modal coupling, and process damping. This indicates that the dynamic model incorporating multiple influencing factors can better reflect the actual milling process. Meanwhile, the results further verify the effectiveness and reliability of the improved FDM proposed in this study for milling stability prediction.

**Figure 20:**
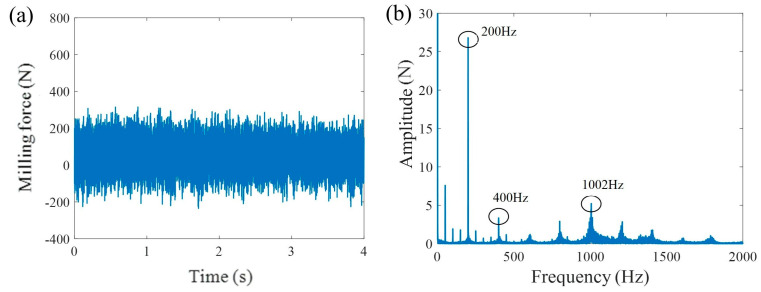
Milling force under No. 3: (**a**) time-domain signal; (**b**) frequency-domain signal.

**Figure 21:**
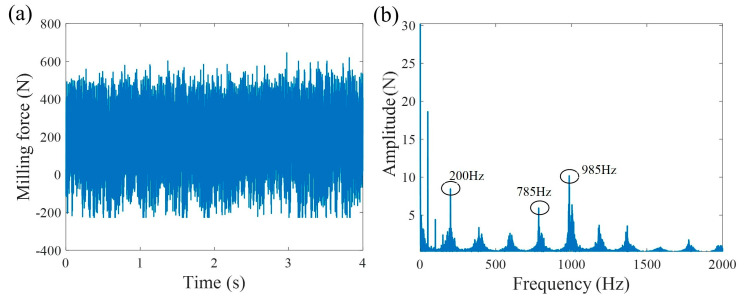
Milling force under No. 4: (**a**) time-domain signal; (**b**) frequency-domain signal.

**Figure 22:**
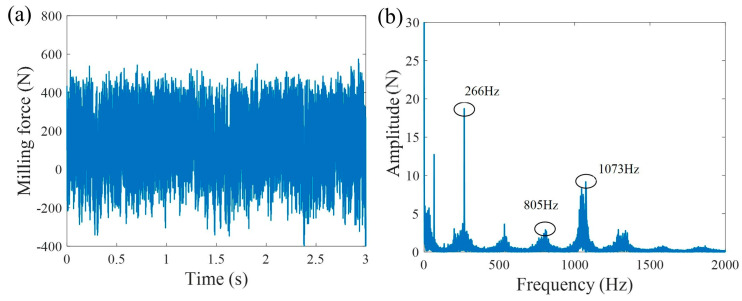
Milling force under No. 8: (**a**) time-domain signal; (**b**) frequency-domain signal.

**Figure 23:**
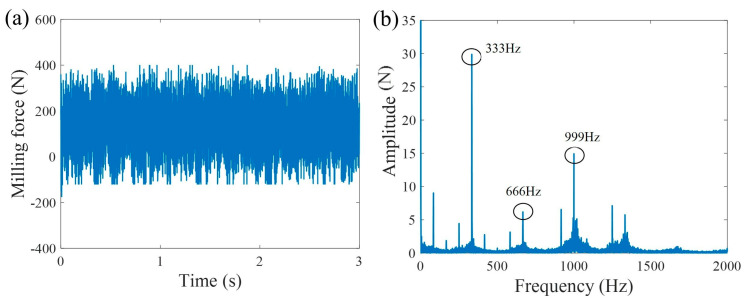
Milling force under No. 10: (**a**) time-domain signal; (**b**) frequency-domain signal.

To further elaborate on the milling experimental results, the measured signals for four typical cutting conditions (Test Nos. 3, 4, 8, and 10) are analyzed, with the corresponding milling force responses presented in Figure 20, Figure 21, Figure 22 and Figure 23. The force signals of Test Nos. 3 and 10 are relatively smooth, and their dominant frequency components are strictly harmonics of the spindle rotational frequency, namely (200 Hz, 400 Hz, 600 Hz) and (333 Hz, 666 Hz, 999 Hz), respectively. In contrast, Test Nos. 4 and 8 exhibit distinct extra chatter frequencies outside the spindle harmonic components, i.e., (785 Hz, 985 Hz) and (805 Hz, 1073 Hz). The frequency differences between these chatter components are 200 Hz and 267 Hz, which are approximately multiples of the corresponding spindle rotational frequencies. These phenomena confirm that Test Nos. 3 and 10 correspond to stable cutting states, whereas Test Nos. 4 and 8 belong to unstable cutting states, which is highly consistent with the theoretical prediction results.

## 6. Conclusions

This study focuses on the regenerative chatter problem in cast iron milling and proposes an improved full-discretization method (FDM) for milling stability analysis. By systematically optimizing the interpolation orders of the state term, time-delay term, and periodic coefficient term, the developed method achieves substantial improvements in both prediction accuracy and computational efficiency. The main contributions of this work are summarized as follows:

(1) A multi-factor coupled dynamic milling model is established, and the optimal interpolation order configuration for FDM is determined. For the milling dynamic model that integrates the regenerative effect, modal coupling effect, and process damping effect, single-factor parametric analysis is conducted to explore the influence of different interpolation orders. The results demonstrate that superior convergence performance and stability boundary prediction accuracy can be realized by adopting second- or third-order interpolation for the state term, third-order interpolation for the time-delay term, and first-order interpolation for the periodic coefficient term. This conclusion corrects the conventional misconception that higher interpolation orders always guarantee better numerical performance.
(2) A novel 321-FDM is proposed, and its comprehensive performance superiority is verified through numerical comparison. Based on the optimal interpolation configuration, the 321-FDM is constructed with third-order interpolation for the state term, second-order interpolation for the time-delay term, and first-order interpolation for the periodic coefficient term. Numerical validation on a single-degree-of-freedom milling system indicates that compared with the traditional semi-discretization method and existing high-order FDMs, the proposed 321-FDM presents a faster convergence rate and lower numerical error under identical discretization intervals. Under typical working conditions, its local discretization error is reduced by an order of magnitude compared with benchmark methods, realizing an optimal balance between computational efficiency and prediction accuracy.
(3) Systematic experiments validate the engineering applicability of the proposed method. Combining hammer-impact modal testing and slot-milling calibration experiments, the practical dynamic parameters and cutting force coefficients of the cast iron milling system are accurately acquired. Twenty groups of milling experiments covering four axial depth-of-cut levels (2–5 mm) and five spindle speed levels (3000–8000 rpm) are conducted based on the stability lobe diagram predicted by the 321-FDM. The consistency between the measured cutting force signals, spectral characteristics, and theoretical prediction results fully verifies the reliability of the multi-factor coupled dynamic model and the effectiveness of the improved FDM for practical milling stability prediction.

## Figures and Tables

**Table 1:** Interpolation combination of state item.

Group	State Term Order	Delay Term Order	Periodic Term Order	FDM
I	1	1	1	111-FDM [10]
II	2	1	1	211-FDM [11]
III	3	1	1	311-FDM [15]
IV	4	1	1	411-FDM
V	1	1	1	111-FDM [10]
VI	1	2	1	121-FDM
VII	1	3	1	131-FDM
VIII	1	4	1	141-FDM
IX	1	1	1	111-FDM [10]
X	1	1	2	112-FDM
XI	1	1	3	113-FDM
XII	1	1	4	114-FDM

**Table 2:** Parameters of single-DOF milling system [34].

Parameter Name	Parameter Symbol	Parameter Value
Natural frequency (Hz)	*ω_n_*	922
Damping ratio (%)	*ζ*	1.1
Modal mass (kg)	*m_x_*	0.03993
Cutting force coefficients (N/m^2^)	*K_tc_*	6 × 10^8^
	*K_rc_*	2 × 10^8^
Number of teeth	*N_t_*	2

**Table 3:** Interpolation combinations of FDM.

Group	State Term Order	Delay Term Order	Periodic Term Order	Corresponding FDM
XI	2	2	1	221-FDM [12]
XII	2	3	1	231-FDM
XIII	3	2	1	321-FDM
XIV	3	3	1	331-FDM

**Table 4:** Model of experimental equipment and tools.

No.	Item	Model
1	Vertical CNC machining center	DNM-415
2	Dynamometer	Kistler
3	Impact hammer	model LC02-C200110185
4	Signal acquisition system	DH5902N
5	Accelerometer	PCB 352A24-SNLW371836
6	Milling cutter	GM-4EL-D12
7	Workpiece	Cast iron part

**Table 5:** Parameters of milling cutter.

Diameter (mm)	Number of Teeth	Length (mm)	Flute Length (mm)	Helix Angle (°)
12	4	100	30	35

**Table 6:** Modal parameters of milling cutter system.

Direction	Natural Frequency (Hz)	Damping Ratio
XX	1343.20	0.0235
XY	1274.15	0.0312
YX	1328.42	0.0272
YY	1566.12	0.0599

**Table 7:** Machining parameters for cutting force coefficient calibration experiments.

No	Spindle Speed (rpm)	Axial Depth of Cut (mm)	Radial Depth of Cut (mm)	Feed Rate (mm/min)
1	2000	1	12	160
2	2000	1	12	320
3	2000	1	12	480
4	2000	1	12	640
5	2000	1	12	800

**Table 8:** Machining parameters for milling experiments.

No	Spindle Speed (rpm)	Axial Depth of Cut (mm)	Radial Depth of Cut (mm)	Feed Rate (mm/min)
1	3000	2	12	0.2
2		3		
3		4		
4		5		
5	4000	2		
6		3		
7		4		
8		5		
9	5000	2		
10		3		
11		4		
12		5		
13	6000	2		
14		3		
15		4		
16		5		
17	8000	2		
18		3		
19		4		
20		5		

## Data Availability

The original contributions presented in this study are included in the article. Further inquiries can be directed to the corresponding author.
